# Cone-Beam Computed Tomography (CBCT) Analysis of Mandibular Condyles’ Diameters in Patient with Juvenile Idiopathic Arthritis and Temporomandibular Joint Affection: A Cross-Sectional Investigation

**DOI:** 10.3390/jcm13175104

**Published:** 2024-08-28

**Authors:** Margherita Donelli, Valentina Lanteri, Alessandro Ugolini, Alessandro Bruni, Paolo Cressoni, Andrea Abate, Cinzia Maspero

**Affiliations:** 1 Department of Biomedical Surgical and Dental Sciences, University of Milan, 20122 Milan, Italy; margherita.donelli@unimi.it (M.D.); paolo.cressoni@gmail.com (P.C.); 2 Fondazione IRCCS Cà Granda, Ospedale Maggiore Policlinico, 20122 Milan, Italy; 3 Surgical, Medical and Dental Department, University of Modena and Reggio Emilia, 41121 Modena, Italy; valentina.lanteri@unimore.it (V.L.); alebruni@unimore.it (A.B.); 4 Department of Sciences Integrated Surgical and Diagnostic, University of Genova, 16126 Genoa, Italy; alessandro.ugolini@unige.it

**Keywords:** malocclusion, juvenile idiopathic arthritis, temporomandibular joint arthritis, CBCT, mandibular condyle

## Abstract

**Objectives:** The aim of this study was to evaluate through analysis using CBCT the transverse and axial diameters of the mandibular condyles in subjects affected by juvenile idiopathic arthritis (JIA) and compare them with those of healthy subjects. **Methods:** The study was conducted on CBCT scans from the digital archive of the Department of Biomedical Surgical and Dental Sciences, University of Milan, including patients with JIA and using healthy subjects as controls. Inclusion criteria: aged between 7 and 25 years old at the time of the CBCT examination; Caucasian ethnicity; diagnosis of JIA according to the International League of Associations for Rheumatology (ILAR) criteria documented in patients’ records; TMJ involvement; good quality CBCTs covering our region of interest (ROI), from the glabella to the mandibular inferior border; no previous orthodontic/orthopedic treatment; no history of craniofacial trauma or congenital birth defects involving the craniofacial area. Each CBCT scan underwent examination using 3Diagnosys^®^ software. Since data were normally distributed, parametric tests were used for analysis. The sample was divided into three groups: (1) bilateral JIA subjects, (2) unilateral JIA subjects, and (3) healthy controls. **Results:** We found a statistically significant reduction (*p* < 0.0001) in the transverse diameter (TR-Diam) of the affected condyles by an average of 1.7 mm, while the axial diameter (AX-Diam) again showed a slight reduction, on average by 0.1 mm, with a non-statistically significant value. Another comparison was made between the unaffected condyles of patients with unilateral JIA and the healthy condyles of the control group. The unilateral unaffected condyles were found to be slightly smaller than those of healthy patients, but without statistically significant differences. We found that in both JIA males and females, the condylar growth tends to stop earlier than the healthy ones. **Conclusions:** The transverse diameter was found to be more affected than the axial one, causing typical bone resorption and condylar shape. Moreover, we showed that the pathology, in the case of unilateral JIA, does not compromise only the affected condyles; the corresponding condyle that seems to be healthy is actually partially compromised. In addition, we observed that the growth of affected condyles of JIA subjects tends to stop earlier than the condyles of the healthy controls.

## 1. Introduction

Juvenile idiopathic arthritis (JIA) is one of the most frequent rheumatic diseases occurring in childhood and adolescence [1]. JIA is defined by the International League of Associations for Rheumatology (ILAR) [2] as a chronic inflammatory condition that affects children under the age of 16, with symptoms lasting over six weeks in the same joint(s) and excluding other diagnoses. Arthritis is characterized by fatigue and fever, joint inflammation, stiffness, swelling and/or painful limited movement [3,4]. 

JIA includes several sub-classifications: systemic-onset arthritis (including joint inflammation along with fever, rash, and inflammation of internal organs), oligoarthritis (affecting four or fewer joints, usually larger ones like the knees), polyarthritis (involving five or more joints and can affect both small and large joints; rheumatoid factor positive and negative), psoriatic arthritis (associated with psoriasis, a skin condition), enthesitis-related arthritis (involving inflammation where tendons and ligaments attach to bones, often associated with spinal inflammation), and undifferentiated arthritis (does not fit into any of the above categories) [5,6].

The overall incidence rates and prevalence vary from 1.6 to 23/100,000 and from 3.8 to 400/100,000. JIA affects females more often than males (with a ratio of 2:1). Oligoarthritis is the most frequent form (incidence rate 3.7 and prevalence 16.8/100,000) [7]. 

Temporo-mandibular joint (TMJ) involvement is observed in 40% to 90% of cases depending on subtype and diagnostic method [4,7,8,9]. TMJ arthritis can lead to pain, functional limitations, and severe complications such as condylar resorption, facial deformities (e.g., for example, reduced posterior facial height, asymmetry of the lower face, micrognathia, and retrognathia), and impaired occlusion (e.g., anterior open bite) [10,11,12].

Early diagnosis and treatment of TMJ arthritis is critical to prevent irreversible damage to the condylar bone and subsequent facial abnormalities [13]. 

Significantly, the articular surface of the condylar head serves as the main site for mandibular growth, making the temporomandibular joint (TMJ) especially vulnerable to damage at the bone surfaces [14,15]. 

However, diagnosing TMJ involvement in young children with JIA can be challenging due to overlapping symptoms with temporomandibular disorders (TMD) and the discrepancy between subjective symptoms and imaging findings [15,16,17].

Clinical examination recommendations for children with JIA include assessing pain location, TMJ tenderness, mandibular deviation, and mouth opening capacity. Imaging techniques such as panoramic dental X-rays, CT, CBCT, and contrast-enhanced MRI play pivotal roles in diagnosing and monitoring TMJ arthritis, with MRI being particularly effective for detecting active synovitis in the early stages [18]. Firstly, the panoramic dental X-ray is a cost-effective and readily available technique. However, it has methodological limitations, such as distortion and highlighting bone alterations only at advanced stages of bone involvement [16]. Development of novel applications of technology offers the way for earlier diagnosis and more targeted available treatments [19]. In some cases, computed tomography (CT) or cone beam computed tomography (CBCT) can be considered as alternatives. Even if contrasted-enhanced MRI is considered the gold standard for soft tissues and is excellent for monitoring active synovitis and preventing structural damage, it fails to provide sufficient information regarding bony structures; CBCT offers an accurate representation of the TMJ and joint space, without the distortions typical of conventional 2D radiology [16,20]. It is particularly sensitive in detecting small bony structure alterations, enabling early diagnosis of degenerative joint disease [21,22,23] CBCT and MRI are widely used for early detection and follow-up of disease activity in JIA patients, with CBCT providing a detailed representation of the TMJ and joint space [5].

The aim of this study was to evaluate through the analysis of CBCT the transverse and axial diameters of the mandibular condyles in subjects affected by JIA (both unilateral and bilateral) and to compare them with those of healthy subjects. This comparison helps to assess the extent to which JIA can compromise the growth and development of condylar dimensions. Additionally, the study analyzed how the pathology presents and changes across different ages and genders among the affected subjects.

## 2. Materials and Methods

### 2.1. Study Design

The present cross-sectional study has examined CBCT scans from participants with juvenile idiopathic arthritis (JIA) and from healthy ones for the controls. 

Each patient received and signed a written informed consent form prior to the collection of medical history data, permitting the anonymous use of the data for scientific purposes.

The study protocol was approved by the competent Institutional Review Board (IRB) as part of the Research Protocol of Fondazione IRCCS Cà Granda Ospedale Maggiore Policlinico. Operative Unit 420, Current Research N. 1, year 2022.

The research adhered to the Declaration of Helsinki guidelines for human studies, maintaining high ethical standards throughout the process.

### 2.2. Type of Participants and Inclusion Criteria

The following retrospective analysis was conducted on CBCT scans from the digital archive of the Department of Biomedical Surgical and Dental Sciences, University of Milan, including patients with unilateral or bilateral JIA and using healthy subjects as controls.

For JIA patients, only the ones who adhered to the following inclusion criteria were included in the study: aged between 7 and 25 years old at the time of the CBCT examination; Caucasian ethnicity; skeletal class II and hyperdivergent; diagnosis of JIA according to the International League of Associations for Rheumatology (ILAR) criteria documented in patients’ records; TMJ involvement; good quality CBCTs covering our region of interest (ROI), from the glabella to the mandibular inferior border; no previous orthodontic/orthopedic treatment; no history of craniofacial trauma or congenital birth defects involving the craniofacial area; and no other pathology affecting bone tissue metabolism.

Just under one-third of oligoarticular and polyarticular JIA patients exhibited a moderate or severe class II skeletal pattern.

For the control group, we selected Caucasian subjects aged between 7 and 22 years old at the time of CBCT examination, skeletal class II and hyperdivergent, with physiological condylar morphology and symmetrical mandibular growth and without any JIA diagnosis or pathological conditions or malformations of the craniofacial area and any familiarity with or history of rheumatic disease. 

At the time the CBCT radiological examination was performed, none of the patients had orthodontic appliances, ensuring that the evaluation was not influenced by potential corrections from the appliances themselves.

In all JIA patients, the pathology onset occurred before the age of 16, and at the time of collecting the CBCT radiological exams, the patients were aged between 7 and 25 years old. 

This made it possible to observe the changes in condylar dimensions throughout the growth period, thus we could assess if and what alterations JIA can cause over time. 

The final sample consisted of 112 patients with JIA and TMJ involvement, including 46 with unilateral JIA (30 females with a mean age of 11.8 ± 2.5 years and 16 males with a mean age of 13.6 ± 1.1 years) and 66 with bilateral JIA (41 females with a mean age of 10.9 ± 2.1 years and 25 males with a mean age of 11.4 ± 2.5 years). The control group included 47 healthy subjects (25 females with a mean age of 11.9 ± 2.3 years and 22 males with a mean age of 11.8 ± 2.1 years). 

The control group’s CBCT scans were conducted for various reasons, such as assessing complex tooth extraction, germectomy of the third molars, impacted teeth, studying the upper airway for ear, nose, and throat (ENT) purposes, and planning complex orthodontic treatments requiring 3D cephalometry (e.g., virtual planning for the insertion of temporary anchorage devices, TADs).

### 2.3. CBCT Examination and Postprocessing 

CBCT images were acquired for all patients using the i-CAT FLX unit from Imaging Sciences International, Inc. CT Dent Ltd. (London, UK). (https://ct-dent.co.uk/i-cat-vision/2012/05/03 (accessed on 21 August 2024)), with consistent exposure parameters. Scans were set for 360° rotation, 300 image frames, 120 kVp, 5 mA, 3.7 s, voxel size of 0.4 mm, and a field of view (FOV) of 16 × 8 or 16 × 11 mm to minimize radiation exposure. The resulting CBCT scans were saved as DICOM files (Digital Imaging and Communications in Medicine).

Each CBCT scan underwent examination using 3Diagnosys^®^ 4.0 software (https://www.exelambulatori.it/wp-content/uploads/2013/07/3DIAGNOSYS_4 (accessed on 21 August 2024)) to measure the axial and transverse mandibular condyles diameters.

### 2.4. Anatomical Analysis on CBCTs

**Figure 1:**
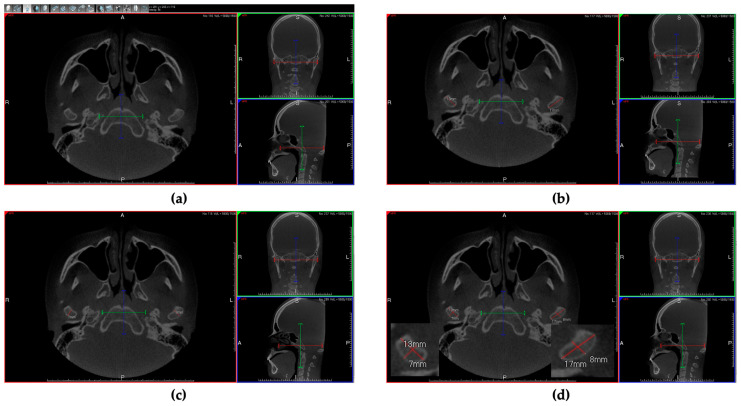
Example of the measurement’s protocols taken to assess the axial (AX_Diam) and transverse (TR_Diam) diameters: (**a**) slice of CBCT in which the condyle had its greatest transverse diameter in both coronal and axial projections; (**b**) considering the anterior and posterior poles of the condyle, the distance between these two points was measured by determining the length of the axial diameters of both condyles; (**c**) considering the medial and lateral poles of the condyle, the distance between these two points was measured by determining the length of the transversal diameters of the two condyles; (**d**) representation of both AX_Diam and TR_Diam of both right and left condyles.

Regarding the transverse condyle’s diameter, we took the slice on the CBCT in which the condyle had its greatest transverse diameter in both coronal and axial projection. Then, exclusively considering the axial projection, two points were taken corresponding to the lateral and medial pole of the mandibular condyle, thus measuring the distance between them (Figure 1a,b,d).

Regarding the axial diameter of each condyle, the procedure of selection of the slice was the same as the previous measures; however, in this case two points were taken on the anterior and posterior surface of the condyle, to outline the axial diameter and thus measure its value (Figure 1c,d).

Finally, the following data were collected and placed in an Excel table:

- Patient’s ID;
- Gender;
- Date of birth;
- Date of CBCT (to determine the age at the time of the examination);
- Classification into bilateral JIA (**Bil_JIA**), unilateral JIA (**Uni_JIA**) or healthy (**Ctrl**) subjects;
- Classification into affected (**Uni_Aff**) or unaffected (**Uni_UnAff**) condyle for unilateral JIA group;
- Classification into right and left condyle for bilateral (**Bil_R; Bil_L**) and control (**Healthy_R; Healthy_L**) groups;
- Right transverse diameter (**TR_R_Diam)**;
- Left transverse diameter (**TR_L_Diam**);
- Right axial diameter (**AX_R_Diam**);
- Left axial diameter (**AX_R_Diam**).

### 2.5. Statistical Analysis and Sample Size Calculation 

A priori sample size calculation was conducted with G*Power (version 3.1.9, http://www.psychologie.hhu.de/arbeitsgruppen/allgemeine-psychologie-und-arbeitspsychologie/gpower.html (accessed on 21 August 2024)) using as reference values those reported by Cavagnetto et al. [12]. On this basis, 23 subjects per group were computed to be needed to reject the null hypothesis that the population means of the case and control groups are equal with probability (power) 0.95. The Type I error probability associated with this test of this null hypothesis is 0.05.

Normal distribution of data was assessed using the Shapiro–Wilk test. Since data were normally distributed, parametric tests were used for analysis. The sample was divided into three groups: (1) bilateral JIA subjects, (2) unilateral JIA subjects, and (3) healthy control subjects. 

First, a paired *t*-test was used for an intra-group analysis; we compared the diameters of the affected condyles (**Uni_Aff**) of unilateral JIA patients with the corresponding unaffected sides (**Uni_UnAff**). 

Then, an independent sample *t*-test was used for an inter-group analysis; we compared the diameters of all affected condyles (**Uni_Aff; Bil_R; Bil_L**) of the JIA patients with the healthy condyles of the controls (**Healthy_R; Healthy_L**). The unaffected condyles of the unilateral JIA group (**Uni_UnAff**) and the healthy ones of the control group (**Healthy_R; Healthy_L**) were compared using an independent *t*-test. 

Finally, a multiple regression model was used to evaluate differences in the condylar growing time in patients with and without JIA, considering subgroups of subjects of the same age and gender.

The significance level for both *t*-tests and multiple regression models was set at *p* < 0.05.

The entire statistical analysis was performed using Stata 17 software (StataCorp. 2021, College Station, TX, USA).

### 2.6. Method Error 

Dahlberg’s formula [24] was utilized to assess the method error, while the intra-class correlation coefficient (ICC) was used to evaluate intra- and inter-operator reliability. All CBCT scans were acquired by the same dental radiologist, and the measurements were performed by a single investigator (M.D.). After a two-week interval, 20 CBCT scans were randomly selected and re-evaluated by a second investigator (A.A.). Subsequently, the same 20 CBCT scans were re-evaluated by the first investigator (M.D.) to assess inter-observer and intra-observer reliability. Both investigators were blinded to the patients’ identities.

## 3. Results

The ICC results reported high agreement for all the measurements; for AX_Diameter, the average (±SD, range) intra-operator and inter-operator ICC values were 0.92 (±0.04, 0.95–0.96) and 0.89 (±0.09, 0.88–0.93) respectively, and for TR_Diameter, the average (±SD, range) intra-operator and inter-operator ICC values were, respectively, 0.95 (±0.04, 0.91–0.93) and 0.88 (±0.08, 0.87–0.89).

The random error calculated for AX_Diameter measurements was about 0.19 mm, and for the TR_Diameter it was about 0.17 mm. Overall, the method error was considered negligible.

### 3.1. Sample Analysis

Table 1 presents the clinical and demography characteristics of the 159 subjects enrolled in the study. Of the 112 subjects with JIA, 89 are female (79.5%) and 23 male (20.5%), of which 66 are affected by bilateral JIA (58.9%) and 46 by unilateral JIA (41.1%). Meanwhile, of the 47 healthy patients, 27 are female (57.4%) and 20 male (42.6%), aged between 7 and 22 years, with class II dental and skeletal structures. In the 66 patients with bilateral JIA, the right condyle was predominantly affected in 25 cases (37.9%) and the left condyle in 14 cases (21.2%). Meanwhile, in the 46 patients with unilateral JIA, the right condyle was affected in 17 cases (36.5%) and the left condyle in 29 cases (63.5%).

### 3.2. Condylar Diameter Results

Descriptive statistics and statistical comparisons for diameter measurements between affected (**Uni_Aff**) and unaffected (**Uni_UnAff**) condyles of unilateral JIA subjects are presented in Table 2. A statistically significant difference (*p* < 0.0001) in the transverse diameter (**TR_Diam**) was found, with an average reduction of 1.5 mm for the affected condyles compared to healthy ones. 

The axial diameter (**AX_Diam**) was also reduced by an average of 0.35 mm, but without a statistically significant difference.

The comparison between the affected condyles (**JIA**) of both unilateral (**Uni_Aff**) and bilateral JIA (**Bil_R; Bil_L**) groups and the healthy condyles of the control group (**Ctrl** = **Healthy_R + Healthy_L**) is reported in Table 3. We found a statistically significant reduction (*p* < 0.0001) in the transverse diameter (**TR-Diam**) of the affected condyles by an average of 1.7 mm, while the axial diameter (**AX-Diam**) again showed a slight reduction, on average by 0.1 mm, with a non-statistically significant value.

Another comparison was made between the unaffected condyles (**Uni_UnAff**) of patients with unilateral JIA and the healthy condyles of the control group (**Ctrl** = **Healthy_R + Healthy_L**); the data collected are reported in Table 3. The unilateral unaffected condyles were found to be slightly smaller than those of healthy patients, but without statistically significant differences.

**Figure 2:**
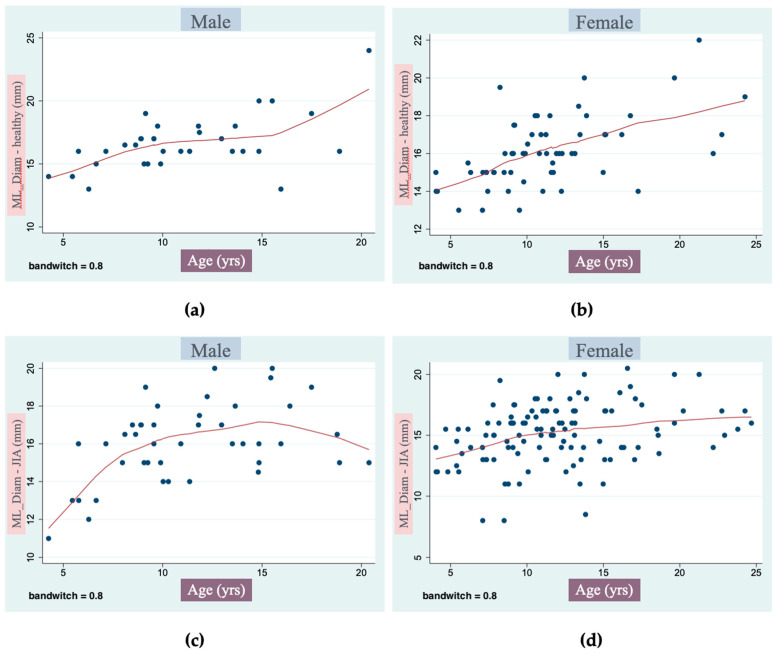
Representation of the different trends of growth of the transverse condylar diameter (the most affected dimension) at different ranges of age in: (**a**) healthy males, (**b**) healthy females, (**c**) JIA males and (**d**) JIA females. As can be seen from the graphs, JIA patients are found to have smaller condyles in their transverse size at the same age and to have an earlier growth cessation compared to healthy condyles.

Finally, through a multiple regression analysis, we assessed at what age the condylar growth rate slows down in males and females and whether there is a difference between healthy and JIA subjects. Analyzing the condyles of subjects at the same age and gender, we found that in both JIA males and females, the condyles were smaller than the ones of the healthy subjects. Moreover, the condylar growth of the patients affected by JIA tends to stop earlier than the healthy ones (Figure 2). 

## 4. Discussion

In JIA patients, it is important to begin treatment early by identifying the disease. Indeed, it is often asymptomatic, leading to delayed treatment [25,26]. Arthritis of the TMJ, if not early diagnosed and treated properly, may lead to bone abnormalities and osseous deformation of the condyle, from minimal erosion to complete destruction of the condylar head. This impaired growth can cause dysmorphic facial features [27,28,29].

The condyle represents one of the major growth centers of the mandible [13]. A thin layer of fibrocartilage separates it from the glenoid fossae, making mandibular growth vulnerable to arthritic changes in JIA patients [30]. This may cause mandibular asymmetry due to the different degrees of progression in articular damage and varying growth impairments between the condyles.

To recognize abnormalities of the shape and size of the mandibular condyles in children of all ages, it is necessary to understand their physiological anatomy through cross-sectional diagnostic imaging [29,31,32]. 

In the literature, only a few articles can be found on the physiological size and shape of the mandibular condyle in children; these studies investigated the articular eminence, temporal fossa, differences between pediatric and adult TMJ, and growth-related remodeling of the TMJ in adults [29,33,34,35]. 

Karlo C.A. et al. (2010) [29] calculated the ratio between the antero-posterior (axial) and medio-lateral (transverse) diameters in childhood growth in healthy subjects and showed that the shape of the mandibular condyles changes from round into oval.

Moreover, to the best of our knowledge, there is little literature regarding the size and shape of the mandibular condyle in subjects affected by JIA. Most research is based on 2D analysis. In fact, some studies have shown that panoramic radiography can detect TMJ destruction and mandibular asymmetries, thus monitoring JIA [36,37], but CBCT (cone beam computed tomography) allows for more detailed 3D measurements. With this method, volumetric alteration of the affected condyles and surrounding structures in patients with unilateral and bilateral JIA can be observed [28,31,38,39,40,41].

Recently, Cavagnetto et al. [12] three-dimensionally evaluated volumetric changes of the different mandibular segments using CBCT in patients affected by juvenile idiopathic arthritis. They saw that the affected condyle of unilateral JIA patients showed statistically significant lower volumes in the hemimandible, in the condyle, and in the ramus. The largest total mandibular volume was observed in the control group, followed by the unilateral JIA group and the bilateral JIA group [42,43,44]. 

Hisie YJ et al. (2020) observed that JIA causes a reduction in mandibular size on the affected side, retrusion of the chin, and prominence of the cheekbone [30,45].

In line with the literature, our results demonstrate that the mandibular condyles are subject to significant changes in size. In particular, the transverse diameter tends to reduce more compared to the axial one; both in intra-group (i.e., between controls condyles and affected condyles of unilateral JIA group) and inter-group analysis (i.e., between affected condyles of unilateral and bilateral JIA subjects and healthy condyles of the control group). Moreover, unaffected unilateral JIA condyles still had smaller dimensions compared to the control ones, demonstrating that the disease globally affects joint growth. 

Finally, as seen in the present study, the disease influences growth, as condylar growth in affected patients stops earlier compared to healthy patients. 

### Limitations and Strengths of the Study

In regard to the limitations of this study, no differences based on subtype of JIA were examined (whether systemic onset arthritis, oligoarthritis, polyarthritis, psoriatic arthritis, enthesitis-related arthritis or undifferentiated arthritis). Moreover, differences in the onset of the disease at TMJ level were not considered. In fact, in patients with bilateral JIA, the temporal differences of the involvement of the two condyles were not known, thus establishing the possibility that the disease did not occur simultaneously in both joints. A longitudinal study would be a valuable tool for analyzing and monitoring the condylar growth. However, frequent exposure to ionizing radiation (CBCT) in growing subjects would be a risk and is contraindicated according to guidelines [46].

Otherwise, regarding the strengths of the research, we showed a high level of reliability (both intra and inter-observer greater than 0.90) for each variable considered. To minimize the possibility of bias, the authors chose to include a control group within the sample that had the same craniofacial characteristics as those patients with JIA and TMJ involvement (class II, hyperdivergent). Indeed, as reported by Von Bremen et al. [47], patients with JIA frequently present a class II high-angle skeletal sagittal relationship. Without this selection, it would have been difficult to determine whether the differences between JIA and non-JIA groups were related to rheumatic condition or different craniofacial characteristics.

## 5. Conclusions

In this retrospective study, we observed that JIA significantly alters condyles. Particularly, the transverse diameter was found to be more affected than the axial one, causing typical bone resorption and condylar shape.

Moreover, we showed that the pathology, in the case of unilateral JIA, does not only compromise the affected condyles; the corresponding one that seems to be healthy is actually partially compromised. In fact, comparing the latter with the condyles of healthy patients showed that the healthy condyles of unilateral JIA patients have a reduced size compared to the condyles of the healthy subjects. 

In addition, it was observed that the growth of affected condyles of JIA subjects tends to stop earlier than the condyles of the healthy ones. 

Thus, it has been shown that JIA impairs the growth and development of condylar dimensions no matter whether the condyles are affected unilaterally or bilaterally.

## Figures and Tables

**Table 1:** Demographic and clinical characteristics of the final sample, with corresponding percentages. For classification of the affected side, in bilateral JIA condyles we considered the most compromised condyle.

Sample Characteristics	Gender		Age (yrs)		Affected Condyle	
	Male [N (Rate%)]	Female [N (Rate%)]	Male Mean Age ± SD	Female Mean Age ± SD	Right [N (Rate%)]	Left [N (Rate%)]
Total (N = 159)	43 (27.0%)	116 (72.9%)				
JIA Group (N = 112)	23 (20.5%)	89 (79.5%)	12.5 ± 1.8	11.4 ± 2.3		
Unilateral JIA (N = 46)	16 (34.8%)	30 (65.2%)	13.6 ± 1.1	11.8 ± 2.5	17 (36.5%)	29 (63.5%)
Bilateral JIA (N = 66)	25 (37.9%)	41 (62.1%)	11.4 ± 2.5	10.9 ± 2.1	25 (37.9%)	14 (21.2%)
Control Group (N = 47)	20 (42.6%)	27 (57.4%)	11.8 ± 2.1	11.9 ± 2.3		

**Table 2:** Descriptive statistics and paired *t*-test between affected (Uni_Aff) and unaffected (Uni_UnAff) sides in unilateral JIA subjects.

Paired *t*-Test	Uni_JIA		Δ(Uni_Aff − Uni_UnAff)	*p* Value
	Uni_Aff	Uni_UnAff		
	Mean ± SD	Mean ± SD	Mean ± SD	
AX_Diam (mm)	7.15 ± 1.38	7.5 ± 1.56	−0.35 ± 1.65	0.1596
TR_Diam (mm)	14.54 ± 2.62	16.04 ± 2.36	−1.5 ± 2.34	0.0001 *

* *p* value < 0.05 was considered as statistically significant.

**Table 3:** Descriptive statistics and two-tailed independent *t*-test between affected condyles of JIA patients (JIA = Uni_Aff + Bil_R + Bil_L) and helathy condyles of the control group (Ctrl); moreover, it was compared the Unaffected condyles (Uni_UnAff) of unilateral JIA subjects with the healthy condyles of the control group (Ctrl).

Two-Tailed Indipendent Sample *t*-Test	JIA *n* = 112	Ctrl *n* = 47	Uni_UnAff *n* = 46	Δ[JIA − Ctrl]		Δ[Uni_UnAff − Ctrl]	
	Mean ± SD	Mean ± SD	Mean ± SD	Mean ± SD	*p* Value	Mean ± SD	*p* Value
AX_Diam (mm)	7.5 ± 1.56	7.40 ± 1.06	7.5 ± 1.56	−0.1 ± 0.004	0.7003	−0.1 ± 0.05	0.7296
TR_Diam (mm)	14.88 ± 2.53	16.56 ± 1.45	16.04 ± 2.36	1.68 ± 0.2	0.0000 *	0.52 ± 0.1	0.2019

* *p* value < 0.05 was considered as statistically significant.

## Data Availability

The data underlying this article will be shared on reasonable request to the corresponding author.
